# Assessment and management of shoulder pain at primary care level

**DOI:** 10.4102/safp.v63i1.5279

**Published:** 2021-03-08

**Authors:** Ntambue Kauta, Elma de Vries, Jean-Pierre du Plessis, Ben Grey, Cameron Anley, Basil Vrettos, Robert Dachs, Stephen Roche

**Affiliations:** 1 Department of Orthopaedic Surgery, Faculty of Health Sciences, University of Cape Town, Cape Town, South Africa; 2 Department of Family Medicine, Faculty of Health Sciences, University of Cape Town, Cape Town, South Africa; 3 School of Public Health, Faculty of Health Sciences, University of Cape Town, Cape Town, South Africa; 4 Department of Orthopaedic Surgery, Faculty of Health Sciences, Stellenbosch University, Stellenbosch, South Africa

**Keywords:** shoulder pain, shoulder stiffness, primary care, guidelines, traumatic shoulder pain, spontaneous shoulder pain, referred shoulder pain

## Abstract

Most patients with shoulder pain will initially visit their community health centre, private general practitioner or family physician, with various levels of experience in the assessment and management of shoulder conditions. Shoulder conditions will range from early, simple ailments that can be treated in the primary care setting, to post-traumatic injuries and complex pathologies requiring the expertise of an orthopaedic surgeon or a fellowship-trained shoulder surgeon. Correct assessment of the patient’s shoulder condition at the index consultation is a prerequisite for appropriate management. This article sets out straightforward guidelines to help general practitioners confidently identify the patient’s source of shoulder pain and initiate an appropriate management plan at primary care level. Criteria for urgent and elective referral for specialist care are also outlined.

## Introduction

Shoulder pain is the third most common musculoskeletal complaint in the primary care setting. Approximately 1% of adults consult general practitioners for a new shoulder complaint annually.^1^

Making a correct assessment of the patient’s shoulder condition at the index consultation is a prerequisite for an appropriate management plan.

It has been shown that for multifactorial reasons the undergraduate training in orthopaedic and musculoskeletal conditions inadequately prepares general practitioners for the management of these conditions.^2^

It is therefore necessary to develop, and regularly update, clear and concise guidelines to help the practising physician confidently and correctly identify the source of shoulder pain and initiate a management plan in the primary care setting. These guidelines should also include criteria for urgent and non-urgent referral for specialist care.

The guidelines set out in this article have been developed based on the available literature on shoulder pain in the primary care setting and expert opinions from the authors.

## Guidelines

Two key points in the initial diagnostic approach to shoulder pain should be to decide whether the patient’s pain is acute or chronic and whether the onset is traumatic or spontaneous.

Acute shoulder pain presents within hours or days (24–72 h) and is often post-traumatic, inflammatory or infectious in origin.

Chronic shoulder pain and stiffness are hallmarks of a wide variety of shoulder pathologies. The multitude of physical tests taught in medical school for the assessment of chronic shoulder pain, whilst useful in narrowing down the diagnosis, may not be able to confirm a specific diagnosis in isolation.^3^

For this reason, these guidelines group painful shoulder pathologies into four broad categories:

- traumatic shoulder pain (history of a fall, motor vehicle accident, assault, sports injury, etc.)
- spontaneous shoulder pain with no red flag symptoms
- shoulder pain associated with red flag symptoms (fever, mass or tumour on the shoulder, loss of weight associated with limited range of motion)
- shoulder pain with a history of prior shoulder surgery (patient has had surgery on that shoulder before).

Each one of these four broad categories will be managed differently, and referral patterns will differ.

### Traumatic shoulder pain (TSP)

The assessment of such pain aims to exclude or diagnose:

- a fracture or dislocation around the shoulder girdle
- a traumatic tear of the rotator cuff tendons
- an injury to the brachial plexus
- an injury to peripheral nerves around the shoulder.

These injuries will need referral to an orthopaedic surgeon. The assessment at primary level will include a history taking, physical examination and radiographic assessment.

At least three radiographic views are required to exclude shoulder fractures or dislocations (antero–posterior, lateral and modified axillary views).

**FIGURE 1:**
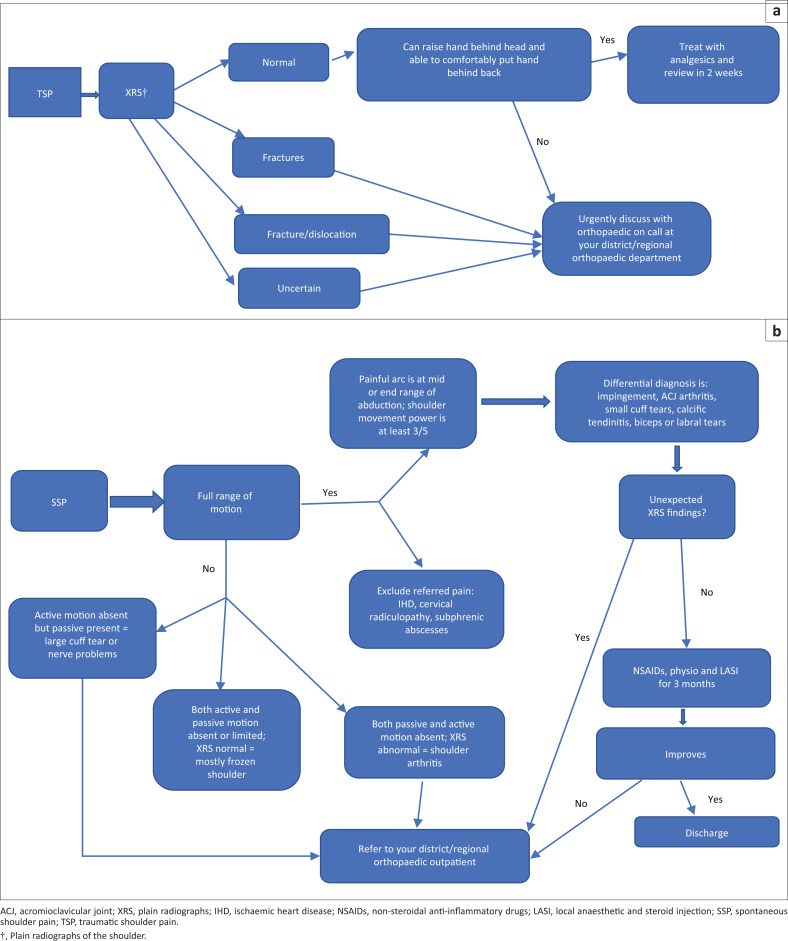
(a) Assessment and management of traumatic shoulder pain. (b) Assessment and management of spontaneous shoulder pain with no red flags.

Figure 1a summarises the assessment and management of traumatic shoulder pain.

### Spontaneous shoulder pain (SSP) with no red flags

The aim of the assessment of spontaneous shoulder pain with no red flags is to differentiate the group of degenerative shoulder conditions, where the initial management may be similar, from referred pain to the shoulder, which requires clinical attention to be directed either to the spine (cervical radiculopathy), chest (ischaemic heart disease) and sometimes the abdomen (subphrenic pathologies). Shoulder pathology that falls into this group includes:

- subacromial impingement
- calcific tendinitis
- chronic rotator cuff tendon tear
- acromioclavicular joint arthritis
- glenohumeral joint arthritis
- frozen shoulder (FS).

The initial management of various degenerative shoulder conditions may include up to a 3-month course of anti-inflammatory and analgesic drugs and physiotherapy. If there is deterioration or no improvement during this initial course of treatment, the treating primary care physician may upscale to local anaesthetic and steroid injections.

Figure 1b summarises this exercise.

### Shoulder pain with red flags

Clinical red flags include:

- fever
- loss of weight
- previous or current history of cancer
- local shoulder warmth
- localised or diffuse swelling around the shoulder.

Such cases should raise the suspicion of a shoulder joint infection or a malignant lesion of the shoulder girdle and must be urgently (same day) referred to a secondary or tertiary level of care. Biological and imaging studies will be undertaken to determine the patient’s potentially life-threatening disease at specialist level.

### Shoulder pain with a history of prior shoulder surgery (patient has had surgery on that shoulder before)

Where an obvious pathology such as a draining sinus is seen (in periprosthetic infections) or a periprosthetic fracture is diagnosed, the patient must be urgently referred to the orthopaedic surgeon.

In a scenario where pathology is less obvious, the physician needs to assess the extent of functional impairment in the shoulder.

If more than 50% of the patient function is maintained, the patient should be treated symptomatically and referred to the orthopaedic surgeon on an elective basis.

If shoulder function has deteriorated to less than 50%, the patient should be referred to a higher level of care on an urgent basis.

### A note on shoulder stiffness

Shoulder stiffness is defined as a loss of active and passive range of motion of the shoulder. Most degenerative shoulder conditions will present with various degrees of stiffness in one or two planes of movement. Shoulder stiffness in all planes of movement is the main clinical feature in advanced glenohumeral arthritis, where early referral is indicated, but it is also the main clinical feature in FS, which should be treated initially in the primary care setting.

Frozen shoulder is a diagnosis of exclusion. It commonly affects patients in the 40–60 age group, females more than males and is also associated with other conditions such as diabetes, thyroid disorders and Parkinson’s. Diabetic patients develop the most severe form of the disease.^4,5^

An initial period of shoulder pain will be substituted by stiffness. Patients will lose external rotation first, and range of motion in all planes will be lost in the late stages.

Shoulder plain radiographs must be normal for a diagnosis of a FS to be made. This underscores the importance of obtaining plain radiographs of the shoulder when assessing chronic shoulder conditions.

Untreated FS stiffness may not spontaneously fully recover as previously taught.^6^

**FIGURE 2:**
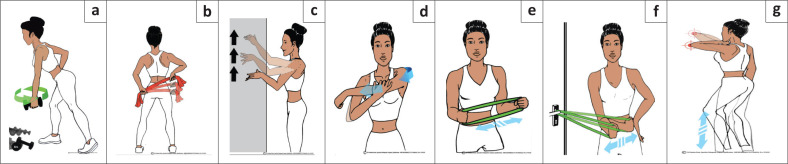
Home-based programme for shoulder stretching and strengthening exercises. (a) Pendulum stretch exercise: Perform 10 revolutions in each direction, once a day. Increase the stretch by holding a light weight (1 kilograms [kg] – 2 kg) in the swinging arm. (b) Towel stretch exercise: One end of a long enough towel is held behind your back in your affected hand and grab the opposite end with your other hand. Hold the towel in a horizontal position. Use your good arm to pull the affected arm upward to stretch it. Perform this stretch 15–30 times once a day. (c) Finger walk exercise: Reach out and touch the wall at waist level with the fingertips of the affected arm. With your elbow slightly bent, slowly walk your fingers up the wall, until you have raised your arm as far as you comfortably can. Slowly lower the arm (with the help of the good arm, if necessary). Perform this exercise 15–30 times a day. (d) Cross-body stretch: Use your good arm to lift your affected arm at the elbow, bring it up and across your body, and apply gentle pressure to stretch the shoulder. Hold the stretch for 15–20 s, 15–30 times per day. (e) Outward strengthening: Strengthening begins once range improves. Hold a rubber exercise band between your hands with your elbows at a 90-degree angle close to your sides. Rotate the lower part of the affected arm outward 15–20 degrees, and hold for 5 s. Repeat 15–20 times, once a day. (f) Inward strengthening: Hook an elastic loop band onto a closed door handle. Hold the other loop end of the elastic band with your hand on the affected side with your elbow flexed 90 degrees and arm at your side. Pull the band inward toward your tummy as far as possible and hold for 5 s. Repeat this 15–20 times, once a day. (g) Armpit stretch/elbow walk: Reach out and touch the wall at waist level with the flexed elbow of the affected arm. Slowly walk your elbow up the wall, until you have raised your arm as far as you comfortably can. Your elbow tip should be doing the work, not your shoulder muscles. Slowly lower the arm (with the help of the good arm, if necessary), and repeat. Repeat this exercise 15–30 times a day.

Patients are encouraged to do a structured home-based physiotherapy programme (Figure 2).

The greatest improvement in range of motion is seen within the first few months after a structured home physiotherapy programme.^7^ A local anaesthetic and steroid injection may be considered to facilitate physiotherapy.

A referral to an orthopaedic or shoulder surgeon should be considered if there has been no improvement in range of motion after 3 months of a home physiotherapy programme.

## Conclusion

At primary care level, an appropriate management plan for shoulder pain can be initiated without a definitive diagnosis, depending on the appropriate categorisation of the patient’s shoulder pain.

These guidelines will help the primary care physician classify shoulder pain into one of the four categories and apply a correct management plan, including referrals to higher level of care.
